# Clinically Apparent and Occult Metastasized Seminoma: Almost Indistinguishable on the Transcriptional Level

**DOI:** 10.1371/journal.pone.0095009

**Published:** 2014-05-01

**Authors:** Christian G. Ruf, Matthias Port, Hans-Ulrich Schmelz, Walter Wagner, Felix Müller, Sven Senf, Cord Matthies, Bertram Müller-Myhsok, Viktor Meineke, Michael Abend

**Affiliations:** 1 Department of Urology, Federal Armed Forces Hospital, Hamburg, Germany; 2 Bundeswehr Institute of Radiobiology, Munich, Germany; 3 Department of Hematology, Hemostasis, Oncology and Stem Cell Transplantation, University Hospital Hannover, Hannover, Germany; 4 Department of Urology, Federal Armed Forces Central Hospital, Koblenz, Germany; 5 Max-Plank-Institute of Psychiatry, Munich, Germany; CCR, National Cancer Institute, NIH, United States of America

## Abstract

**Purpose:**

The aim of the present study was to examine the biological differences between seminomas with occult and clinically apparent metastases at the time of diagnosis of the primary tumor to gain insight into the biology of these tumors and facilitate the identification of novel predictors of seminoma metastasis.

**Materials and Methods:**

Total RNA including small RNAs was isolated from testicular tumors of patients with pure seminoma presenting with lymphogenic metastasis (n = 5, clinical stage IIb/c) and occult metastasis (n = 5, clinical stage I). The regulation of biological processes was examined (1) throughout the mRNA transcriptome (whole genome microarrays, 8×60 K Array, Agilent with 4 samples/group) and (2) the miRNA transcriptome employing small RNA next generation sequencing (SOLID, Life Technologies with 5 samples/group). Protein coding genes (mRNAs) and small RNAs showing a significant (≥2-fold) difference between the groups were identified. Finally (3), we examined 95 candidate miRNAs in 36 apparent metastasized and another 5 occult metastasized seminoma using logistic regression analysis.

**Results:**

Among 19,596 genes, on average 12,894 mRNAs appeared expressed (65.8%, SD+/−2.4; range, 62.0–69.3%) and 16.99×10^6^/13.94×10^6^ small RNA reads were identified for apparent/occult metastasized seminoma. These reads on average convert into 9,901/9,675 small RNAs including 422/404 mature microRNAs. None of these mRNAs/small RNAs met our selection criteria for candidate genes. From 95 candidate miRNAs 44 appeared expressed, with 3 of them showing weak but significant (p = 0.05) differences among both groups.

**Conclusions:**

Occult and apparent metastasized seminomas are biologically almost indistinguishable and probably represent no separate tumor entities. These findings may simplify future research on seminoma metastasis.

## Introduction

Testicular tumor is the most common tumor among young men and is associated with a 5-year survival rate of approximately 100% in the early stages. Pure seminoma is currently the most frequent histological subtype (55%) and in up to 70% of cases, it presents without visible metastasis at primary staging [1], [2]. Clinical stage I (cS I) patients without metastases are cured by orchiectomy alone. However, despite modern staging and classification procedures, up to 30% of cS I seminoma patients bear occult metastasis in primary staging and relapse after orchiectomy [1], [3]. To date, no reliable biological parameter or alternative predictor exists to differentiate occult metastasized stages (metastasis detected during follow-up) from non-metastasized seminoma. The identification of patients with occult metastasis is important to prevent toxicity (e.g., cardiovascular and kidney disease, secondary malignancies and decreased fertility) caused by unnecessary adjuvant treatment or diagnostic procedures during follow-up [4].

Recent studies propose the existence of certain risk factors associated with both, apparent and occult seminoma metastasis. For instance, multivariate analyses showed that large tumor size (>6 cm) and infiltration of the rete testis are risk factors associated with clinically apparent metastasis [5] and occult metastasis from seminoma [1], [3], [6]–[8].

Considering these similarities, we hypothesized that primary tumors with clinically apparent metastases and those with occult metastases might share a considerable number of biological characteristics (namely the process of metastasis). If seminomas with apparent and occult metastasis do not represent different metastatic subtypes, this would simplify future studies considerably, because we would only have to discriminate metastasized seminoma from non-metastasized seminoma without considering subtypes of metastasis. Investigation at the molecular level appear promising: a study from our group showed that clinical risk factors discriminated metastasized from non-metastasized seminomas in approximately 65% of cases [8], whereas transcriptional gene expression changes discriminated up to 88% of cases, which reflects the value of molecular biological examinations [9], [10]. Furthermore, several promising biomarkers of metastatic spread [11], [12] and potential serum biomarkers of malignant germ cell tumors such as SNPs [13] have been identified in addition to the miRNA 371–73 cluster and miRNA 302 [14], [15]. This again underlines the potential of molecular biological markers and the need to carefully examine biological processes associated with apparent and occult metastasis from seminoma.

In the present study, we investigated differences in the regulation of biological processes at the mRNA and miRNA transcriptional level between seminomas with occult and those with apparent metastases. As a first approach we performed a whole genome microarray analysis to screen for genome-wide mRNA transcriptional gene expression changes. As a second approach whole genome changes of all small RNAs were assessed by next generation sequencing (NGS). In previous analysis we identified miRNAs which completely discriminated non-metastasized from metastasized seminoma (accepted for publication). As a third approach we used 95 from these miRNA species and examined their potential to discriminate apparent metastasized seminoma (n = 36) from occult metastasized seminoma (n = 5) using qRT-PCR.

## Materials and Methods

### 1. Patient Selection

Patients in both groups comprising occult metastasis seminoma (n = 2×5) presented without visible metastasis at primary staging, received no adjuvant treatment, and developed retroperitoneal tumors during the 2-year follow-up. Patients in both groups comprising apparent metastasis at primary staging (n = 5 and n = 36) were limited to those with clinical stage IIb and IIc to include lymphogenic metastatic spread only and to provide a high level of diagnostic accuracy (avoiding questionable lymph nodes).

### 2. Tissue Samples and Histological Examination

Samples from testicular tumor biopsies were incubated in RNA-later solution (Qiagen, Hilden, Germany) immediately after collection and later stored at −20°C. All tissue samples were examined by an experienced pathologist for histological and TNM classification (table 1). The local ethics commission of the medical council of Hamburg approved the study and all human samples were collected after obtaining written informed consent.

**Table 1:** Descriptive statistics of patients and tumor characteristics are shown for those (A) employing whole genome microarray (mRNA) and NGS (small RNAs including miRNAs) and (b) using qRT-PCR for selected 95 different miRNAs.

metastasis subtype	descriptive statistic	age at diagnosis (years)	tumor size (mm)	initial clinical stage
**A) whole genome microarray and NGS analysis**				
detectable	mean	45,2	37,8	cSIIb-c
	stdev	10,8	14,9	
	min	31,6	12,0	
	max	60,7	50,0	
	n	5		
occult	mean	32,1	38,6	cSI
	stdev	5,4	16,2	
	min	23,4	18,0	
	max	36,6	55,0	
	n	5		
	p-value	0,04	0,9	
**B) qRT-PCR**				
detectable	mean	37,4	49,4	cSIIb-c
	stdev	7,2	35,1	
	min	21,1	10,0	
	max	51,6	180,0	
	n	36		
occult	mean	45,4	45,8	cSI
	stdev	4,5	21,0	
	min	38,0	24,0	
	max	51,0	80,0	
	n	5		
	p-value	0,02	0,8	

P-values were calculated using either the t-test or a non-parametric Mann-Whitney rank sum test where applicable.

### 3. RNA Isolation

Tissues frozen in RNA-later solution (Qiagen, Hilden, Germany) were carefully thawed, homogenized (Homogenizer, Omni, Warrenton, USA) and digested using proteinase K. Total RNA including small RNAs was isolated (mirVana Kit, Life Technologies, Penzberg, Germany) and the remaining DNA was digested. RNA was stored at −80°C until use. The quality and quantity of isolated total RNA were measured spectrophotometrically (NanoDrop, PeqLab Biotechnology, Erlangen, Germany), and RNA integrity was assessed by the 2100 Agilent Bioanalyser (Life Science Group, Penzberg, Germany). Only RNA specimens with a A260/A280 ratio ≥2.0 (Nanodrop) and RNA integrity number (RIN)≥8.0 were used for whole genome microarray or small RNA Next Generation Sequencing (IMGM Laboratories, Martinsried, Germany/CeGat, Tübingen, Germany) or qRT-PCR.

### 4. Whole Genome Microarray and Data Analysis of mRNAs

Genome-wide expression profiling was performed using the Agilent oligo microarray GE 8×60 K (Agilent Technologies, Waldbronn, Germany) combined with a one-color based hybridization protocol for 4 samples per group. In brief, total RNA was reverse transcribed into cDNA, converted to cyanine-3-labeled-cRNA (Quick Amp Labeling Kit One-Color, Life Technologies), purified, fragmented and hybridized on the microarray. Signals on the microarray were detected using the Agilent DNA Microarray Scanner. GeneSpring GX12 software was used to quantile normalize the raw data. Gene expression (quantile normalized log2-transformed probe signals) was analyzed as an outcome and compared between the two groups using the non-parametric Welch’s approximate t-test. Genes had to be expressed in >50% of samples per group. Only significant and ≥2-fold differences in gene expression between the two groups were considered for comparisons, and unadjusted p-values and those adjusted for multiple comparisons (false discovery rate) were calculated. All gene candidates underwent gene set enrichment analyses using PANTHER pathway software (http://www.pantherdb.org/), which groups genes with similar biological functions based on their annotation. All data are stored and accessible under the Gene Expression Omnibus platform of NCBI (http://www.ncbi.nlm.nih.gov/geo/query/acc.cgi?acc=GSE55198).

### 5. Small RNA Next Generation Sequencing and Data Analysis

Genome-wide small RNA sequencing was performed using the SOLiD5500xl Next Generation Sequencing Technology (Life Technologies, Penzberg, Germany). In brief, total RNA (5 samples per group) was purified (PureLink miRNA isolation Kit), enriched small RNAs were ligated to SOLiD adaptors and reverse transcribed (SOLiD RT primer and ArrayScript RT). The cDNA was purified (MinElute PCR purification Kit, Qiagen), a cut-off size of 60–80 nt was selected (Novex pre-cast gel products, Invitrogen), the cDNA was in-gel amplified, and samples were barcoded using a SOLiD 3′Barcode primer at the same time. Amplified cDNA was purified (PureLink PCR Micro Kit, Life Technologies) and used in emulsion PCR (SOLiD EZ Bead). Thereafter, the emulsion was broken to recover enriched beads and the so-called di-base probes were used by the SOLiD system in the sequencing-by-ligation procedure. In addition to the SOLiD5500xl inherent software (LifeScope) used for image and signal processing, the software CLC Genomics Workbench 5.1 (CLC bio) was used for clustering, counting, and annotation of all generated 75 bp reads. After discarding reads without an adaptor sequence and those shorter than 15 bp (trimming), reads were assigned to known miRNAs (Sanger miRBase release 18, http://www.mirbase.org/) and known non-coding RNAs (ensembl database Homo_sapiens.GRCh37.67.ncrna.fa, www.ensembl.org). Small RNAs showing a significant [16] and ≥2-fold difference in gene expression among groups and at least 50 reads were considered for comparisons. These candidate genes were further analyzed based on their ability to discriminate between groups using support vector machines.

### 6. miRNA Measurements using qRT-PCR

A custom made low density array design (96a format) was used to simultaneously detect 95 miRNAs (TaqMan primer probe assays) on a 384-well platform (LDA). A 20 ng RNA aliquot of each RNA sample was reverse transcribed using the TaqMan microRNA Reverse Transcription Kit. 100 µl cDNA (20 ng RNA equivalent) was mixed with 100 µl 2× RT-PCR master mix and pipetted into 2 fill ports of the LDA. Four different samples could be processed per LDA. Cards were centrifuged twice (1,200 rpm, 1 min, Multifuge3S-R, Heraeus, Germany), sealed, and transferred into the 7900 qRT-PCR instrument. The qRT-PCR was run for two hours following the qRT-PCR protocol for 384-well LDA format. Ahead of our experiment we established the upper limit of the linear-dynamic range of our qRT-PCR using replicate measurements on one of our samples. The upper limit occurred at CT≤29. CT values were normalized relative to the median gene expression of the examined microRNAs. Normalized CT values of both groups were examined on their discriminative capability employing logistic regression analysis.

All chemicals for qRT-PCR using TaqMan chemistry were provided by Life Technologies, Darmstadt, Germany. All technical procedures for qRT-PCR were performed in accordance with standard operating procedures implemented in our laboratory in 2008 when the Bundeswehr Institute of Radiobiology became accredited according to DIN EN ISO 9001/2008.

## Results

### RNA Isolation

For whole genome microarray analysis and NGS methodology we isolated on average 38.9 µg of total RNA (SD+/−8.5, range: 8.1–68.8) per 10 mg of biopsy samples. The average RNA integrity number (RIN) was 8.8 (SD+/−0.3, range: 8.3–9.4).

For qRT-PCR analysis we isolated on average 30.6 µg of total RNA (SD+/−11.9, range: 7.8–79.7) per 10 mg of biopsy samples. The average RNA integrity number (RIN) was 8.3 (SD+/−0.3, range: 6.2–9.6). One outlier with RIN 4.6 in additional quality control experiments did show no signs of degradation including the qRT-PCR controls. No DNA contamination could be detected in our samples.

### Whole Genome Microarray and PANTHER Classification

Of 19,596 gene mRNAs (42,545 transcripts) spotted on the whole genome microarray, on average 65.8% (SD+/−2.4, range: 62.0–69.3%) were distinguishable from the background (expressed). The biological replicate samples showed high correlation coefficients (Pearson’s correlation) within and between experimental groups:

R-squared values from 0.912–0.975 within apparent metastasis
R-squared values from 0.938–0.967 within occult metastasis
R-squared values from 0.872–0.978 between the two groups.

No differentially expressed RNAs were identified by applying the stringent approach (2-fold difference in gene expression among groups and p-values corrected for multiple comparisons). Therefore, the non-stringent filtering approach (2-fold difference in gene expression and p-values not corrected for multiple comparisons) was applied. No up-regulated and only 30 down-regulated genes were identified. Median p-values of 0.03 (range: 0.001−0.05) and median fold-changes of 2.7 (SD+/−2.5) with higher fold-changes associated with higher p-values (due to higher variance) suggested that most of these 30 genes met the selection criteria at the border of becoming significant. Because downstream enrichment analysis requires lists of at least 100 differentially expressed genes, to run this analysis we changed our inclusion criteria and generated lists with fold-change cut-offs ≥1.5 and uncorrected p-values ≤0.05 knowing that this would increase the number of false positive candidate genes. The resulting list comprised 149 up-regulated and 132 down-regulated genes. Using this list for downstream enrichment analysis of biological processes, molecular functions, cellular components, protein classes and pathways did not generate any significant enrichment for up- or down-regulated genes.

### Small RNA Next Generation Sequencing

The average total number of reads for apparent/occult metastasized seminoma was 16.99×10^6^/13.94×10^6^ (SD+/−2.35×10^6^/1.99×10^6^) and on average 30.4%/30.1% reads (SD+/−15.3%/14.6%) remained for further analysis after trimming of the reads. Among these reads, 53.3–69.6%/53.2–66.1% appeared as annotated reads with 7.1–11.9%/7.4–13.6% representing annotated small RNAs. These small RNA reads convert to an average number of 9,901/9,675 (SD+/−2,243/1,819, range: 7,335–12,451/8,447–12,789) small RNA species eligible for statistical analysis in apparent/occult metastasized seminoma, respectively. Among these, we counted an average of 422/404 mature microRNAs (SD+/−63.6/30.3, range: 325–491/368–430). None of these small RNAs met our selection criteria for candidate genes.

### miRNA Measurements using qRT-PCR

From 95 miRNAs (with some of them spotted more than once for internal quality control of the LDA technology) altogether 61 miRNAs appeared expressed (CT value ≤29). Of them 44 miRNAs were expressed in at least 50% of the samples and eligible for logistic regression analysis (table 2). Only 3 (miR99a, miR125b-2* and let7a) of them did show statistically significant associations with p-values (p = 0.05) close to become borderline significant and partly calculated based on an even smaller sample size than 5 (n = 3) in the group of occult metastasized seminoma.

**Table 2:** Normalized gene expression (CT-values) of 44 genes expressed in ≥50% of tumor samples per metastasis subtype was compared using descriptive statistic and logistic regression analysis (p-value).

	miR-16	miR-99a	let-7g	mmu-miR-93	rno-miR-7*	miR-664	miR-574-3p	miR-484	miR-4454	miR-4286	miR-423-3P	let-7g	miR-345	miR-331	miR-320
***detectable metastasis***															
Mean	10,6	15,3	14,6	14,2	17,1	16,7	15,0	13,6	10,5	10,8	7,2	14,9	16,1	16,1	13,8
median	10,6	15,5	14,7	14,0	17,0	16,8	14,9	13,5	10,6	10,8	7,3	14,9	16,0	16,2	13,8
stdev	0,7	1,0	0,7	1,0	0,7	0,6	1,0	1,0	0,6	0,8	0,9	0,8	1,0	0,7	0,9
min	9,3	12,3	13,1	11,9	15,8	15,5	12,9	11,4	8,3	8,6	5,1	13,4	13,9	14,8	11,9
Max	13,3	16,9	16,3	16,2	19,0	17,9	16,9	15,1	11,7	12,3	9,0	17,1	18,3	17,7	15,5
n	36	30	34	36	24	31	35	36	36	36	36	35	34	34	36
***occult metastasis***															
mean	10,3	13,7	14,1	14,1	17,2	16,7	14,5	13,7	11,0	10,1	7,0	14,1	15,9	16,6	13,7
median	10,3	13,1	14,1	13,5	17,1	16,7	14,3	13,4	11,0	11,1	7,0	13,9	16,0	16,5	13,8
stdev	0,5	1,3	0,8	1,0	0,5	0,7	1,3	1,0	0,5	2,6	0,3	0,8	0,5	0,3	0,8
min	9,6	12,8	13,2	13,3	16,7	15,7	13,3	12,7	10,4	5,6	6,6	13,4	15,4	16,3	12,4
max	10,9	15,2	15,2	15,6	17,9	17,4	15,9	15,3	11,8	11,8	7,4	15,1	16,4	16,9	14,6
n	5	3	4	5	4	5	4	5	5	5	5	4	5	5	5
p-value	0,4	0,05	0,2	0,8	0,8	0,9	0,5	0,8	0,1	0,2	0,7	0,1	0,6	0,2	0,7
	**miR-30d**	**miR-30b**	**miR-29a**	**miR-25**	**miR-197**	**miR-191**	**miR-182**	**miR-17**	**let-7g**	**miR-15b**	**miR-150**	**miR-145**	**let-7g**	**miR-126**	miR-125b-2*
***detectable metastasis***															
mean	16,5	13,5	15,3	16,0	15,8	13,2	16,4	13,4	14,7	16,1	13,0	13,3	14,6	12,6	16,6
median	16,4	13,6	15,3	15,9	15,9	13,3	16,3	13,3	14,8	16,0	12,6	13,1	14,7	12,4	16,6
stdev	0,5	0,8	0,8	0,9	0,8	0,7	0,9	0,9	0,7	0,7	1,4	1,5	0,7	0,8	0,5
min	15,7	12,0	13,3	14,8	13,9	11,8	14,9	11,8	13,2	14,8	9,7	10,2	13,2	11,4	15,5
max	17,6	14,8	16,7	18,0	17,1	14,6	18,0	15,6	16,8	17,4	15,9	16,4	16,7	14,9	17,6
n	31	36	33	36	36	36	27	36	35	34	36	36	34	36	32
***occult metastasis***															
mean	16,4	12,8	14,8	16,0	15,8	13,3	15,6	12,9	14,2	15,8	13,1	12,7	14,2	12,8	15,9
median	16,4	12,8	14,4	15,1	16,1	13,1	15,6	13,0	14,0	16,3	12,4	12,9	14,1	12,8	16,2
stdev	0,7	0,3	1,2	1,5	0,5	0,7	1,0	0,9	0,5	0,8	2,0	1,7	0,9	0,9	0,9
min	15,3	12,5	13,6	14,6	15,1	12,6	14,6	11,5	13,8	15,0	11,5	10,4	13,4	11,4	14,5
max	17,3	13,4	16,8	18,1	16,2	14,3	16,7	13,8	14,9	16,4	16,0	14,6	15,2	14,0	16,7
n	5	5	5	5	5	5	4	5	4	5	4	5	4	5	5
p-value	0,6	0,1	0,3	0,9	0,9	0,9	0,2	0,3	0,2	0,5	0,9	0,4	0,3	0,6	0,05
	**miR-106b**	**let-7g**	**let-7b**	**let-7a**	**miR-371-3p**	**miR-372**	**miR-373**	**miR-302a**	**miR-302b**	**miR-302d**	**miR-367**	**miR-200c**	**miR-222**	**miR-16**	
***detectable metastasis***															
mean	16,7	14,6	15,5	16,7	14,7	10,2	15,6	15,5	15,5	16,3	16,0	15,6	12,8	10,6	
median	16,6	14,7	15,7	16,8	14,9	9,9	15,2	15,6	15,4	16,1	16,0	15,6	12,7	10,5	
stdev	1,0	0,8	0,9	0,7	1,1	1,7	1,2	1,0	1,1	0,9	0,9	1,2	1,0	0,8	
min	15,0	13,0	12,7	14,9	12,8	7,9	13,8	12,8	13,3	14,8	14,4	12,6	10,8	9,5	
max	19,1	16,7	16,9	18,1	17,7	16,1	18,9	17,1	17,3	18,3	17,5	17,9	15,4	13,4	
n	27	34	31	23	35	36	35	31	30	31	24	29	36	36	
***occult metastasis***															
mean	15,4	14,0	15,0	15,6	14,7	11,3	15,3	15,1	15,6	15,7	16,0	16,2	13,0	10,4	
median	15,5	13,9	14,9	15,7	14,7	9,7	15,3	15,6	15,7	15,9	15,9	16,2	13,2	10,2	
stdev	0,8	0,9	1,3	0,2	0,4	3,7	0,2	1,6	0,8	0,8	1,4	0,8	1,4	0,7	
min	14,6	13,2	13,8	15,3	14,2	9,4	15,1	13,4	14,5	14,8	14,7	15,3	11,2	9,8	
max	16,1	15,1	16,6	15,7	15,1	17,9	15,6	16,4	16,4	16,3	17,4	17,1	15,0	11,6	
n	3	4	4	3	4	5	4	3	4	3	4	4	5	5	
p-value	0,08	0,2	0,3	0,05	0,9	0,3	0,6	0,6	0,9	0,3	0,9	0,4	0,6	0,6	

## Discussion

In the present study, we examined biological differences between seminomas with clinically apparent metastasis at the primary staging and those without (occult). Whole genome screening for protein coding genes detected approximately 66% of all known genes. Further analysis of these 12,894 genes using our stringent selection criteria showed no differentially expressed genes, and only 30 down-regulated genes were identified using uncorrected p-values. Those genes appeared as weak candidates based on their fold-change differences and the p-values close to 0.05, in addition to the bias associated with false positive results due to the p-values not corrected for multiple comparisons. Therefore, they were considered questionable candidate genes. To detect changes in gene expression associated with the regulation of different biological processes, we used a less stringent filtering process to identify candidate genes for PANTHER analysis, keeping in mind that the candidate RNAs identified by this filtering approach must be interpreted with caution. Despite this modification, no biological processes, molecular functions, cellular components, protein classes or pathways appeared significantly over or underrepresented in the PANTHER analysis.

We also examined approximately 9,800 small RNAs, including 413 mature microRNAs, and no significant changes in gene expression were observed between the two metastasis subtypes.

With a third approach we increased the sample size (n = 41) and focused on those 95 miRNAs which according to recent work significantly discriminate metastasized from non-metastasized seminoma (accepted for publication). However, when using these genes to discriminate apparent from occult metastasized seminoma we only found weak associations with p-values close to 0.05 for three miRNAs. These p-values were partly calculated based on a small sample size (n = 3) in the group of occult metastasized seminoma and, therefore, should be judged cautiously.

These similarities in the regulation of biological processes at the mRNA as well as the miRNA transcriptome suggest that the two metastasis subtypes do have more features in common (e.g. process of metastasis and corresponding gene expression regulation) than differing from each other.

Our findings are also in agreement with prior clinical and epidemiological findings showing that metastasis incidence is positively associated with an increased size of the primary tumor [5]–[8], [17], [18]. This association can be interpreted differently. Assumed biology of occult metastasis would differ from apparent seminoma metastasis (e.g., decreased proliferation or increased apoptosis) this could explain the delayed detection of occult metastasis up to 2 years after the diagnosis of the primary tumor. However, our results suggest that in growing primary tumors that are diagnosed late, the process of metastasis may already be in progress, and the metastases as well as the primary tumor are growing. A delay in the staging procedure (e.g., CT scan) increases the likelihood of occult metastases reaching a clinically apparent size while the primary tumor is getting larger as well, resulting in the conversion of later occurring occult metastases into apparent metastases, with both sharing the same biological processes. This interpretation is supported by the fact that other risk factors for apparent metastasis from seminoma, such as infiltration of the rete testis and the tunica albuginea, the infiltration of blood and lymphatic vessels and the level of certain tumor markers [5], [8] have been validated as risk factors for apparent and occult metastasized seminoma in prior studies [6], [7], [17]. Hence, our data imply that apparent and occult metastases from seminoma do not have to be treated as two separate tumor entities, which may simplify future studies.

Another important factor is the identification of parameters capable of discriminating metastasized from non-metastasized seminoma. In this regard, modifications at the mRNA and miRNA transcriptome are superior to already identified clinical or epidemiological risk factors [8], [10]. In the present study, we showed that both metastatic subtypes behave in a similar manner with regard to control of biological processes and are almost indistinguishable from each other. This again implies that changes at the regulatory level can be used to distinguish metastasized from non-metastasized seminoma without the burden of considering apparent/occult metastasis as two separate entities.

The present study has several limitations. The number of biopsies examined for whole genome transcriptome changes was low. However, with the screening methodologies used, false positive and false negative results are a bigger concern than low power: from a methodological and statistical point of view, there is a greater likelihood of “seeing more” than of overlooking associations, in particular in the absence of adjustment for multiple comparisons as in the present study. In our experience, the use of whole genome microarrays is associated with a rate of false positive/negative results of approximately 20% [19]. However, even with unadjusted p-values, we did not detect any differences in the control of biological processes between the two metastatic subtypes. This verifies our findings despite the low number of biopsies, and indicates that the results are not likely to change regardless of the number of biopsy samples analyzed.

In order to increase the tumor sample size we performed a third examination comprising 95 genes (miRNAs) and 41 samples with 5 samples originating from patients who decided in favor of surveillance and developed a later metastasis. Still, the additional number of occult metastasis employed was only 5, but these patients are rare when bearing in mind that it represents the output of collected samples from 3 testis cancer centers over the last 7 years.

In conclusion, seminomas with occult metastasis and those with apparent metastasis at the time of the diagnosis of the primary tumor do have more features in common than differing from each other, thus making them almost indistinguishable from the biological point of view. Hence, both metastasis subtypes may not represent separate tumor entities. This will simplify future molecular biological examinations using mRNAs or small RNAs as potential risk factors to discriminate metastasized from non-metastasized seminoma.
